# Suicide by hanging: Results from a national survey in Switzerland and its implications for suicide prevention

**DOI:** 10.1371/journal.pone.0220508

**Published:** 2019-09-18

**Authors:** Thomas Reisch, Chantal Hartmann, Alexander Hemmer, Christine Bartsch

**Affiliations:** 1 Hospital of Psychiatry Muensingen, Department of Psychiatry, Muensingen, Switzerland; 2 Hospital of Psychiatry, Department of Psychiatry, University of Bern, Bern, Switzerland; 3 Alfred Wegener Institute, Helmholtz Association, Department of Infrastructure/Administration / Operations and Research Platforms, Bremerhaven, Germany; Stellenbosch University, SOUTH AFRICA

## Abstract

**Background:**

Hanging is a frequent suicide method, but developing measures to prevent suicide by this method is particularly challenging. The aim of this study is to gain new knowledge that would enable the design of effective of measures that would help prevent suicide by hanging.

**Methods:**

A total of 6,497 suicides registered across the eight Swiss Forensic Institutes (IRM) were analysed. Of these, 1,282 (19.7%) persons hung themselves. T-test and chi-square tests. and chi-square tests were used to analyse …(or determine, or investigate) …group differences regarding sociodemographic variables and triggers

**Findings:**

Men and women who hung themselves showed no significant differences in sociodemographic variables. However, women were significantly more likely to have a psychiatric illness history, whereas men were more likely to have somatic diagnoses. In controlled environments, people used shelves, plumbing and windows more often than beams, pipes, bars and hooks to hang themselves. Compared with other suicide methods, hanging was more likely to have been triggered by partner and financial problems.

**Conclusions:**

Suicide by hanging can be best prevented in institutions (e.g. psychiatric hospitals, somatic hospitals, prisons). These institutions should be structurally evaluated and modified with a primary focus on sanitary areas, windows and shelves. Otherwise, it is important to use general suicide prevention measures, such as awareness raising and staff training in medical settings, low-threshold treatment options and regular suicide risk assessment for people at risk.

## Introduction

Worldwide, hanging is one of the most used suicide methods [1]. In Switzerland it is the most frequent suicide method [2]. The lethality of hanging is between 69% and 84% and thus only slightly lower than the lethality of shooting [3]. In addition, there has been a general increase over time in hanging as a method of suicide [4, 5].

Various studies show that less-educated [6, 7] and unemployed people are more likely to hang themselves [8]. Some studies have shown a connection between hanging and marriage, relationship and financial problems [9, 10]. However, the literature shows conflicting results relating to civil status. Isolated studies have shown that married people hang themselves more often [9], whereas at least one study showed that people in this group hang themselves less frequently [6]. Concerning age, the literature yields similarly heterogeneous results. Whereas a few publications have shown that younger persons tend to hang themselves more often [9], others have found that the middle-aged are more prone to choosing this suicide method [4, 11]. Some papers found that predominantly older people hang themselves [6]. Regarding gender, approximately three-quarters of all people who hang themselves are male [4, 11].

Several publications present significant differences in hanging between men and women. For example, Kanamüller, Riipinenn, Riala, Paloneva and Hakko [12] showed that women who had hung themselves had been psychiatrically hospitalised more often than men who had hung themselves. Accordingly, Kanchan and Menezes [13] argued that statistical analyses in epidemiological studies regarding hanging should address males and females separately.

People who choose hanging as the method of suicide differ from those who choose other methods in a few ways. For instance, De Leo, Evans and Neulinger [14] showed that men who hang themselves are more likely to suffer from a psychiatric illness and less often from a somatic disease compared with men that choose suicide by shooting. Overall, however, only a few comparative studies exist.

In summary, findings regarding sociodemographic and medical risk factors are rare and heterogeneous. Further studies in diverse countries are called for to understand these converse findings better. Our study aims to contribute here.

A number of researchers have suggested that fewer starting points exist for the prevention of suicide by hanging than other methods of suicide [8, 15]. This is partly due to the fact that strangulation tools, such as ropes, are easy to obtain [1, 4, 16, 17, 18] as well as to the fact that many different and readily available low-hanging suspension points can be used [17, 19, 20, 21, 22]. Furthermore, most suicides by hanging take place in a private setting, which reduces the probability of a life-saving intervention [23, 24]. For these reasons, suicide prevention of hanging poses special challenges [1]. Preventing suicide by hanging though is particularly important because of its frequency and lethality [25].

In addition to improvements in overall suicide prevention [12, 23] and influencing social acceptability of the method [6, 18, 26], suicide prevention mainly focuses on the prevention of hanging in controlled environments, such as psychiatric hospitals, police custody, prisons, dormitories and other places where people are closely supervised by third parties [27]. Approximately 10% of all suicides by hanging take place in these high-risk environments [1, 17, 24], and hanging is the most frequently used method of suicide in psychiatric hospitals [21].

The first goal of this study is to improve our comprehension of the medical and sociodemographic variables within the group of people who hang themselves. Further, we search for distinctive features in people that hang themselves in controlled and uncontrolled environments and study the exact details of how they carried out the hangings. Based on our findings, we make suggestions to improve suicide prevention measures.

## Method

Our study is part of the Swiss National Science Foundation Project “Suicides in Switzerland: a detailed national survey of the years 2000 to 2010” (NF 32003b_133070 / 1) [28]. In this project, all suicides examined by Swiss Forensic Institutes (IRM) are included. This dataset allows us to analyse all suicides by hanging between 2000 and 2010. To do this we applied a standardised data entry form developed by the research group [24]. No other data source was used or linked. Detailed information on the data collection can be found in Thoeni, Reisch, Hemmer and Bartsch [29], Ruff, Bartsch, Glasow and Reisch [30] and Gauthier, Reisch and Bartsch [31].

The above-mentioned dataset contains all given suicide cases from the IRM of Zurich, Berne, Basel, Chur, St. Gallen, Lausanne, Geneva and Locarno between the beginning of 2000 to the end of 2010. The data collection by master’s students in medicine took place between spring 2011 and winter 2013. Sociodemographic variables (e.g. age, sex, place of death, partnership, citizenship), various medical variables (e.g. method of suicide, previous suicide attempts, psychiatric history, reference to psychiatric diagnosis) as well as details on suicide (farewell letters, exact location) and applied suicide method (e.g. place of hanging, suspension point) were recorded.

The completed data entry forms were anonymised, scanned and automatically imported into an SPSS file. After this semi-automatic process, the data were manually controlled for scanning or data processing errors. For the present study, we extracted the data regarding suicide by hanging.

### Total sample

The total sample consisted of 6,497 suicides from the years 2000 to 2010. Of these, 4,480 were male (69%) and 2,016 (31%) female. The mean age in the total sample is 50.3 years (*SD* = 18.6 years). After shooting, hanging was the second most common suicide method. Of the total sample of 6,249 suicides, 1,282 people (19.2%) died from suicide by hanging (see Table 1).

**Table 1:** Total Sample: Suicide Method.

Method	Number (%)
Firearms	1338 (20.6)
Hanging	1282 (19.7)
Jumping from Height	1054 (16.2)
Intoxication	983 (15.1)
Train	560 (8.6)
Other	1280 (19.7)
Total	6497 (100.0)

### Analysis

We performed standard statistical analyses (t-tests, chi-square tests and descriptive statistics) using SPSS (version 22) and executed all tests for each gender separately because many studies showed marked differences for male and female subjects. Medical officers of the IRM investigated each suicide, mainly to exclude the possibility of influences of third persons in the deaths of suicide victims. Due to this fact, some of the variables relevant for our research questions were incomplete. The included variables therefore have marked differences regarding missing data. In many files, the psychosocial background was insufficiently mentioned (e.g. in the farewell letters). Due to this, data related to relationship problems as well as financial problems were often able to be included in the analysis.

When comparing hanging to other suicide methods, we carried out two different types of analyses. First, we compared hanging to all other methods of suicide. Secondly, we compared hanging to suicide by shooting, jumping off heights, jumping in front of a train and self-intoxication individually.

## Results

### Sociodemographic data

Of the 1,282 suicides by hanging, 1,008 (78.6%) were male and 274 (21.4%) female. The average age is 49.2 years (*SD* = 18.3 years) and there is no statistically significant age difference between men and women (women 47.6 years, *SD* = 18.3 years; men 49.7 years, *SD* = 18.3 years).

IRM files contain data regarding education for 584 people, with 12.7% holding a university or a college degree. Approximately two-thirds lived in a partnership, one-eighth were unemployed and almost three-quarters were Swiss citizens. We found no statistically significant differences between men and women in respect to these variables (for details see Table 2).

**Table 2:** Sociodemographic comparison of suicides by hanging: Gender-specific results.

	Male		Female		Total		
Variable (missing data sets)	N	%	N	%	N	%	Chi^2^ (p)
Partnership (443)	444	68.3	126	66.7	570	67.9	n.s.
Level of Employment: employed (645):	455	87.8	103	86.6	558	87.6	n.s.
University Degree (698):	63	13.1	11	10.7	74	12.7	n.s.
Nationality: Swiss (204)	606	71.6	172	74.1	778	72.2	n.s.
Prior Attempted Suicide (lifetime) (794)	162	42.7	65	59.6	227	46.5	9.71 (0.002)
Current Psychiatric Inpatient Treatment (661)	60	6	39	14.2	99	7.7	20.73 (< .001)
Prior Inpatient Psychiatric Treatment (661)	97	9.6	48	17.5	145	11.3	13.39 (<0.001)
Psychiatric Outpatient Treatment (661)	130	12.9	61	22.3	191	14.9	14.91 (<0.001)
History of somatic illness (490)	220	22	46	16.9	266	20.9	n.s.
Farewell Letter (0)	430	42.7	113	41.2	543	42.4	n.s.
Trigger: Relationship problems with partner (294)	151	15	38	13.9	189	14.7	n.s.
Trigger: Financial problems (294)	85	8.4	7	2.6	92	7.2	11.17 (0.001)
Psychiatric Illness (0)	483	47.9	164	59.93	647	50.5	12.28 (<0.001)

n.s.: not significant

### Medical data

Almost half of the people who died from suicide by hanging had a previous suicide attempt noted in their biography. This rate was larger for women than for men. Numerically, men hung themselves more often during a psychiatric hospitalisation, but the relative proportion of all suicides by hanging is significantly higher in women. Women also had a higher rate of previous psychiatric in- and outpatient treatment than men. More than one fifth who hung themselves had a known history of a somatic illness. We found relationship crises as triggers, as well as farewell letters similarly often for both sexes. Overall, financial issues as a background to suicides were rare but this was statistically significantly more often in males than in females. Psychiatric problems were indicated in 647 data sets (50.5%), which occurred proportionately less frequently in men than in women (for details see Table 2).

### Details of hanging procedure

In 1,052 cases (82.1% of all records) the strangulation tool is specified in the reported IRM data. Women were significantly more likely to use clothes or accessories (including belts), whereas men numerically more often used ropes.

Women more often hanged themselves in their own home and in a psychiatric hospital, whereas men more often chose public places (e.g. stairwells), nature, their workplace or while in police custody/prison (for details see Table 3).

**Table 3:** Details of suicides by hanging: Gender specific results.

	Total (%)	N Male (%)	N Female (%)	Chi^2^ (p)
**Strangulation Tool (230 missings)**				
Clothing	203 (19.3)	122 (14.8)	81 (36.0)	49.27 (<0.001)
Household	85 (8.1)	62 (7.5)	23 (10.2)	n.s.
Cable	183 (17.4)	147 (17.8)	36 (16.0)	n.s.
Robe	539 (51.2)	460 (55.6)	79 (35.1)	29.79 (<0.001)
Other	42 (4.0)	36 (4.4)	6 (2.7)	n.s.
**Total**	1052 (100)	827 (100)	225 (100)	59.11 (<0.001)
**Place of Hanging (0 missing)**				
Home	851 (66.4)	639 (63.4)	212 (77.4)	18.87 (<0.001)
Public Place	104 (8.1)	94 (9.3)	10 (3.6)	9.31 (0.002)
Nature	94 (7.3)	86 (8.5)	8 (2.9)	9.99 (0.002)
Workplace	54 (4.2)	52 (5.2)	2 (0.7)	10.47 (0.001)
Psychiatry	74 (5.8)	41 (4.1)	33 (12.0)	25.20 (<0.001)
Residential home	18 (1.4)	16 (1.6)	2 (0.7)	n.s.
Police/Prison	76 (5.9)	71 (7.0)	5 (1.8)	10.52 (0.001)
Other	11 (0.9)	9. (0.9)	2 (0.7)	n.s.
**Total**	1282 (100)	1008 (100)	274 (100)	69.02 (<0.001)

n.s.: not significant

The suspension point of the strangulation tool was 250.4 cm on average (*SD* = 131.5 cm). On average, men chose higher suspension points (men: 259.9 cm, *SD* = 137.6 cm; women: 206.8 cm, *SD* = 87.8 cm; *t* = 2.34; *p* = 0.02). In 60.2% (534 of 887 datasets), the feet touched the ground (incomplete hanging entries in 69.2% of the datasets). We found no significant difference between men and women regarding complete versus incomplete hanging (incomplete hanging: males: 59.8%, females: 61.7%).

The exact suspension point was described in 627 datasets (48.9%). Inside homes, people chose a variety of suspension points. with beams, rods/tubes and shelves being the most common points of suspension. If a door was used, in almost half of the cases (18/40; 45%) the strangulation tool was attached to the handle of a door. Outside buildings, trees were used almost exclusively.

In protected environments (e.g. hospitals, prison, police custody, residential homes) people often hanged themselves on furniture, windows and on sanitary installations (shower rod, etc.). Almost half the people outside protected environments used some kind of installation such as pipes, bars, hooks, curtain rails and beams as suspension points. In protected environments, people used trees less often (for details see Table 4).

**Table 4:** Comparison of suspension points in controlled and uncontrolled environments (prison, hospital, residential home).

Variable (missing)	Total (N = 627)	Uncontrolled Environments (N = 541)	Controlled Environments (N = 86)	Chi^2^ (p)
	N (%)	N (%)	N (%)	
**Installation**				
Beam	128 (20.4)	124 (22.9)	4 (4.7)	15.24 (<0.001)
Tube	73 (11.6)	72 (13.3)	1 (1.2)	10.64 (0.001)
Bar	27 (4.3)	25 (4.6)	2 (2.3)	n.s.
**Hook**	34 (5.4)	33 (6.1)	1 (1.2)	n.s.
**Furniture**	57 (9.1)	33 (6.1)	24 (27.9)	42.70 (<0.001)
**Heater**	16 (2.6)	15 (2.8)	1 (1.2)	n.s.
**Window**	44 (7.0)	22 (4.1)	22 (25.6)	52.64 (<0.001)
**Door**				
Door (excl. knob)	29 (4.6)	27 (5.0)	2 (2.3)	n.s.
Doorknob (only)	26 (4.1)	23 (4.3)	3 (3.5)	n.s.
**Sanitary Facility**	27 (4.3)	14 (2.6)	13 (15.1)	28.27 (<0.001)
**Tree**	78 (12.4)	76 (14.0)	2 (2.3)	9.36 (0.002)
**Other**				
Ladder	11 (1.8)	11 (2.0)	0 (0.0)	n.s.
Stairs/Railing	41 (6.5)	38 (7.0)	3 (3.5)	n.s.
Other	36 (5.7)	28 (5.2)	8 (9.3)	n.s.

n.s.: not significant

### Comparison of sociodemographic medical profiles (hanging vs. other methods of suicide)

In comparison with men who committed suicide by all other methods recorded in the dataset, men who hung themselves were less likely to be married (Chi^2^ = 18.06; p< .05, Bonferroni corrected), and to live in a partnership (Chi^2^ = 8.54; p< .05, Bonferroni corrected)) and were less likely to be Swiss (Chi^2^ = 38.45; p< .05, Bonferroni corrected) and less likely had a history of a somatic illness (Chi^2^ = 25.27; p< .05, Bonferroni corrected).

In comparison to all other methods of suicide, women who hanged themselves rarely had a somatic anamnesis (Chi^2^ = 26.78; p< .05, Bonferroni corrected).

### Comparison hanging vs. shooting

Men who hanged themselves were less often Swiss nationals than men who shot themselves. They were more likely to have attempted suicide in the past, had received inpatient psychiatric treatment more often and seldom showed a somatic anamnesis. They less often wrote a farewell letter.

Women who hanged themselves, compared with women who shot themselves, did not show any group difference.

### Comparison hanging vs. suicide by train

Compared with men who died by rail suicide, men that died by hanging were more often married and less often hospitalised for psychiatric treatment. We find indications of financial problems and partnership problems as a trigger more often in men who hanged themselves. They also more often wrote farewell letters.

Women who hanged themselves did not differ from those women who died by rail suicide.

### Comparison hanging vs. jumping from heights

Men who hanged themselves were more often married, less often possessed a university degree, less often had a somatic anamnesis, more often left a farewell letter and we found evidence of relationship problems as well as financial problems as a trigger more often than for men who died by jumping from heights.

Women who hanged themselves had somatic problems less often than women who died by jumping from heights.

### Comparison hanging vs. intoxication

Men who hanged themselves were more likely to be married than men who intoxicated themselves, more likely to live in a partnership, less likely to have been in psychiatric inpatient treatment, were in outpatient psychiatric treatment less often and showed a somatic anamnesis less frequently.

Women who hanged themselves showed similar differences in their profile as evident for men. In addition, the proportion of women being currently (but not lifetime) in psychiatric inpatient treatment at the time of death was higher than for those who intoxicated themselves (for details see Tables 5 and 6).

**Table 5:** Do Women who hang themselves differ from women who used other suicide methods? Comparison of sociodemographic and medical variables.

	Suicide Method					Chi^2^ (p) Group Comparison (Bonferroni corrected)			
	Hanging	Firearms	Train	Jumping	Intoxication	Hanging vs Firearms	Hanging vs Train	Hanging vs. Jumping	Hanging vs. Intoxication
	%	%	%	%	%	Chi^2^ (p)	Chi^2^ (p)	Chi^2^ (p)	Chi^2^ (p)
Married	36	40.6	37.9	34.8	24.5	n.s.	n.s.	n.s.	9.80 (< .05)
Partnership	66.7	76.3	69.1	63.9	53.6	n.s.	n.s.	n.s.	8.61 (< .05)
Unemployed	13.4	9.8	11	8.5	16.8	n.s.	n.s.	n.s.	n.s.
University Degree	10.7	9.8	6	21.6	12.7	n.s.	n.s.	n.s.	n.s.
Nationality Swiss	74.1	83.1	82.2	78.2	80.7	n.s.	n.s.	n.s.	n.s.
Attempted Suicide	59.6	41.9	81	64	70	n.s.	n.s.	n.s.	n.s.
Psychiatric inpatient (currently)	14.2	1.3	21.5	12.4	5	n.s.	n.s.	n.s.	20.51 (< .05)
Psychiatric inpatient (lifetime)	17.5	13.8	21.5	20	25.7	n.s.	n.s.	n.s.	n.s.
Psychiatric outpatient treatment	22.3	21.3	24.2	22.7	32	n.s.	n.s.	n.s.	n.s.
Somatic Anamnesis	33.1	38.6	25.6	52.4	68.8	n.s.	n.s.	12.6 (< .05)	50.35 (< .05)
Farewell Letter	41.2	57.5	32.4	31.5	44.4	n.s.	n.s.	n.s.	n.s.
Trigger: Partnership	13.9	23.8	7.8	8.6	10	n.s.	n.s.	n.s.	n.s.
Trigger: Finance	2.6	6.3	1.8	1.7	3.4	n.s.	n.s.	n.s.	n.s.

n.s.: not significant

**Table 6:** Do men who hang themselves differ from men who used other suicide methods? Comparison of sociodemographic and medical variables.

	Suicide Method					Chi^2^ (p) Group Comparison (Bonferroni corrected)			
	Hanging	Firearms	Train	Jumping	Intoxication	Hanging vs Firearms	Hanging vs Train	Hanging vs. Jumping	Hanging vs. Intoxication
	%	%	%	%	%	Chi^2^ (p)	Chi^2^ (p)	Chi^2^ (p)	Chi^2^ (p)
Married	42.7	41.6	26.4	34.6	25.5	n.s.	22.31 (< .05)	8.54 (< .05).	35.17 (< .05)
Partnership	68.3	65.6	63.6	63.3	51.3	n.s.	n.s.	n.s.	23.98 (< .05)
Unemployed	12.2	8	15.6	11.2	20.8	n.s.	n.s.	n.s.	n.s.
University Degree	13.1	16.3	16.3	25.6	20.8	n.s.	n.s.	16.88 (< .05)	n.s.
Nationality Swiss	71.6	88.9	77.4	78.4	76.5	93.42 (< .05)	n.s.	n.s.	n.s.
Attempted Suicide	42.7	19.7	39.6	45.7	55.7	47.08 (< .05)	n.s.	n.s.	n.s.
Psychiatric inpatient (currently)	6	1.4	11.4	7.3	4.3	36.09 (< .05)	11.27 (< .05)	n.s.	n.s.
Psychiatric inpatient (lifetime)	9.6	4.4	8.8	12.3	18.9	24.66 (< .05)	n.s.	n.s.	25.07 (< .05)
Psychiatric outpatient treatment	12.9	9.8	15	17.8	26.5	n.s.	n.s.	n.s.	41.10 (< .05)
Somatic Anamnesis	42.3	53	38.3	56.6	65.1	13.53 (< .05)	n.s.	16.65 (< .05)	37.35 (< .05)
Farewell Letter	42.7	49.7	30.5	27.8	42.2	11.09 (< .05)	15.76 (< .05)	37.10 (< .05)	n.s.
Trigger: Partnership	15	16.5	7.9	7.7	11.2	n.s.	11.10 (< .05)	19.13 (< .05)	n.s.
Trigger: Finance	8.4	9.9	1.5	2.7	5.2	n.s.	19.86 (< .05)	22.10 (< .05)	n.s.

n.s.: not significant.

## Discussion

Our results regarding suicide method are broadly consistent with other studies. As it is generally the case in Switzerland, the sample examined in our study shows that suicide by hanging is the second most often used suicide method. As with Kurtulus, Nilufer Yonguc, Boz and Acar [11] and Russo, Verzeletti, Piras and De Ferrari [4], approximately three-quarters of those who hanged themselves were male. Slightly over 10% of individuals in the dataset hanged themselves in controlled environments. This corresponds to the range found by other researchers [1, 17, 23, 24]. In the following, we discuss the individual results in detail.

As described in the literature [1, 4, 16, 17], people who hang themselves use readily available tools of strangulation. In the present study, women tended to use clothes, whereas men used ropes.

More than half of the people who hanged themselves touched the ground with their legs. This finding is in line with several other studies [1, 17, 19, 20, 22]. This result underlines that low-level suspension points are used as well [1]. Although most of the persons used a suspension point at or above door handles, a few cases were found below, especially in institutions with high rates of suicide, such as psychiatric hospitals or prisons. Securing these points should be especially considered in suicide prevention in such controlled environments

Hanging requires preparation time. It is obvious that this is exactly what co-determines the choice of place of execution. As also found in other studies [24], most of the individuals studied in the present study hanged themselves in their own homes. Outside of their own premises, a significant proportion of suicides by hanging was in nature. Individuals, as described by Gunnell, Bennewith, Hawton, Simkin and Kapur [1], often used trees as points of suspension. According to Russo, Verzeletti, Piras and De Ferrari [4], a trouble-free preparation and execution of a suicide is easily possible at such locations and thus method-specific suicide prevention is difficult.

Further analysis of the execution locations shows that in controlled environments, such as prisons or closed psychiatric wards, individuals use other kinds of execution locations. It is not surprising that in such environments individuals use trees less, as accessibility to them is restricted for suicidal individuals and accordingly, a preparation in the garden or courtyard of such an institution is usually not possible. Surprisingly, unlike in uncontrolled environments, individuals use typical suspension points, such as pipes and rods, in institutions less frequently [17]. One possible explanation here is that these have already been made inaccessible through structural suicide prevention measures in possible retreat areas. Similar to Glasow [32] and Ruff, Bartsch, Glasow and Reisch [30], we found that individuals used sanitary facilities within controlled environments more often than other areas. Sanitary facilities provide typical retreat areas in controlled environments. Our data show that within the framework of institutional suicide prevention, these retreat areas need to be secured with special care. Indeed, suicide-preventive alternatives are often possible for shower hoses, shower rods, etc. without greater restriction of function and aesthetics.

Within controlled environments, more than one-quarter of cases used furniture (e.g. shelves) as points of suspension. According to our research, this is a result not yet described in the literature. Although it does not directly emerge from this study, we assume that most of the furniture used were in individuals own rooms and cells and not in public areas of these institutions. Technically, it is possible to build e.g. shelves that make hanging significantly more difficult, even impossible. It is also possible e.g. to avoid using shelves completely in these areas. Securing furniture, including shelves in closets, should be an inherent part of structural suicide prevention in the above-mentioned institutions. The same goes for windows, which are also commonly used suspension points in protected environments. Windows should therefore also be examined using a critical suicide prevention perspective and modified accordingly, if necessary.

We found no relevant differences between men and women regarding sociodemographic variables in the method suicide by hanging. The medical variables, in contrast, show distinct differences. Women had more previous suicide attempts noted, and they were hospitalised for psychiatric treatment more often at the time of suicide or had been at an earlier point. Our finding matches Kanamüller’s results (12), which found that women also exhibited more hospitalisations in their anamnesis. Women are thus at least partly (e.g. in the context of a suicide attempt or psychiatric hospitalisation) accessible for general suicide prevention. Considering that in any form of out- or inpatient psychiatric treatment, suicide risk assessment is a standard procedure, no concrete improvements for suicide prevention can be derived from these results.

Women and men who hanged themselves were (numerically) less often Swiss nationals in comparison with persons that died by other suicide methods. In Switzerland, non-Swiss nationals have very limited access to firearms, whereas suicide by firearms is one of the main methods used by Swiss men [29]. In most Western countries, suicide by hanging is more common than in Switzerland. Therefore, on average, suicide by hanging can be considered a rather familiar method in the migrant population group. This difference regarding nationality is therefore primarily explained by the reduced physical availability of firearms and probably also by the greater psychological availability of the method in non-Swiss citizens.

Corresponding with De Leo, Evans and Neulinger [14], we found differences between individuals who died by suicide by hanging and those who used a different method. In both sexes, current relationship problems were (numerically) more prevalent in suicide by hanging than in other methods (except firearms). In addition, men who hanged themselves were (numerically) more often married or lived in a partnership. This result suggests that hanging, which is carried out at home at a greater rate than most other methods, could often have a relationship component, as described by Bastia and Kar [10]. Easier access to family or couple counselling as well as increasing the degree of familiarity of such offers could contribute to suicide prevention.

### Limitations

Our paper has several limitations. The most important limitation of the study relates to the included data. The IRMs do not systematically examine all suicides in Switzerland. The main reason for this is the fact that not all cantons in Switzerland have an IRM and other medical officers examine suicides in some of the Swiss cantons. More importantly, a selection bias could have occurred. The IRM investigate all cases in which third-person influence must be excluded. This is rarely the case in hanging but may be of some significance in other suicide methods (e.g. intoxication). The IRM files are based on data from the police and doctors and the findings from the IRM investigation. The quality and quantity of the data about variables that are of less importance to the IRM’s examination is also significantly lower. In respect to these variables, missing data is significantly higher and the quality of results significantly lower.

### Implications for suicide prevention

Concerning the method of suicide by hanging, prevention is a challenge. However, it can be improved selectively. If the suicide takes place outside of controlled environments, it is often carried out in the person’s own home or in seclusion with the aids for hanging being ubiquitously available. Suicide by hanging can thus be executed relatively quickly, impulsively and invisibly from third parties, even within the person’s own home.

To reduce the number of suicides by hanging, general suicide prevention, for instance regular suicide risk assessments in ambulatory therapy or a high level of awareness raising of 24-hour crisis intervention services, must be continued consistently and even expanded. General programmes that increase the awareness of suicide [33], for example, by staff training programmes focusing on early recognising and dealing with suicide patients can help to reduce suicides.

More suicide prevention options exist within controlled environments. However, we also find a large number of possible strangulation tools and suspension points in these places, which makes structural and method-restrictive interventions difficult. Our results show that sanitary facilities must be secured with greater care. For furniture, and shelves in particular, products must be used that inhibit, or at least impede, hanging. In addition, windows should be secured thoroughly. Since doorknobs are frequently used as suspension points, investments should be made in the development of suicide-proof doorknobs.

Due to the complexity and peculiarity of controlled environments, we recommend suicide prevention assessments by external experts to effectively design in-house structural suicide prevention.

## Supporting information

S1 Appendix SPSS data set. The file (S1_File). contains data that support the presented analyses. Due to data safety reasons the file is anonymized and the variable “age” was reduced to 5-year age groups.
(SAV)

## References

[1] Gunnell D, Bennewith O, Hawton K, Simkin S, Kapur N. The epidemiology and prevention of suicide by hanging: a systematic review. Int J Epidemiol. 2005;34(2):433–42. 10.1093/ije/dyh398 . 15659471

[2] Obsan [Suisse Health Observatory] (n. d.). Suizid [suicide]. Retrieved October 18 2018, from https://www.obsan.admin.ch/de/indikatoren/suizid.

[3] Baker SP, Hu G, Wilcox HC, Baker TD. Increase in suicide by hanging / suffocation in the US, 2000–2010. Am J Prev Med. 2013;44(2):146–9. 10.1016/j.amepre.2012.10.010 . 23332330 PMC3553495

[4] Russo MC, Verzeletti A, Piras M, De Ferrari F. Hanging Deaths: A Retrospective Study Regarding 260 Cases. Am J Forensic Med Pathol. 2016;37(3):141–5. 10.1097/PAF.0000000000000239 . 27281442

[5] Wilkinson D, Gunnell D. Comparison of trends in method-specific suicide rates in Australia and England & Wales, 1968–97. Aust N Z J Public Health. 2000 4;24(2):153–7. 10790934 10.1111/j.1467-842x.2000.tb00135.x

[6] Starkuviene S, Kalediene R, Petrauskiene J. Epidemic of suicide by hanging in Lithuania: does socio-demographic status matter? Public Health. 2006;120(8):769–75. 10.1016/j.puhe.2006.04.009 . 16828493

[7] Monsef Kasmaee V, Zohrevandi B, Asadi P, Shakouri N. Non-judicial hanging in Guilan Province, Iran between 2011 and 2013. Emerg. 2015;3(4):155–8. . 26495406 PMC4608343

[8] Kosky RJ, Dundas P. Death by hanging: implications for prevention of an important method of youth suicide. Aust N Z J Psychiatry. 2000;34(5):836–41. 10.1080/j.1440-1614.2000.00807.x . 11037371

[9] Vijayakumari N. Suicidal hanging: a prospective study. J Indian Academy Forensic Med. 2011;33:355–357.

[10] Bastia BK, Kar N. A psychological autopsy study of suicidal hanging from Cuttack, India: focus on stressful life situations. Arch Suicide Res. 2009;13(1):100–4. 10.1080/13811110802572221 . 19123113

[11] Kurtulus A, Nilufer Yonguc G, Boz B, Acar K. Anatomopathological findings in hangings: a retrospective autopsy study. Med Sci Law. 2013;53(2):80–4. 10.1258/msl.2012.012030 . 23275431

[12] Kanamüller J, Riipinen P, Riala K, Paloneva E, Hakko H. Hanging suicides in northern Finland: a descriptive epidemiological study. Death Stud. 2016;40(4):205–10. 10.1080/07481187.2015.1117537 . 26681439

[13] Kanchan T, Menezes RG. Suicidal hanging in Manipal, South India—victim profile and gender differences. J Forensic Leg Med. 2008;15(8):493–6. 10.1016/j.jflm.2008.05.004 . 18926500

[14] De Leo D, Evans R, Neulinger K. Hanging, firearm, and non—domestic gas suicides among males: a comparative study. Aust N Z J Psychiatry. 2002;36(2):183–9. 10.1046/j.1440-1614.2001.01013.x 11982538

[15] Cantor CH, Baume PJ. Access to methods of suicide: what impact? Aust N Z J Psychiatry. 1998;32(1):8–14. 10.3109/00048679809062700 . 9565178

[16] Bhosle SH, Zanjad NP, Dake MD, Godbole HV. Deaths due to hanging among adolescents—a 10-year retrospective study. J Forensic Leg Med. 2015;29:30–3. 10.1016/j.jflm.2014.11.003 . 25572082

[17] Bennewith O, Gunnell D, Kapur N, Turnbull P, Simkin S, Sutton L, et al Suicide by hanging: multicentre study based on coroners' records in England. Br J Psychiatry. 2005;186:260–1. 10.1192/bjp.186.3.260 . 15738509

[18] Biddle L, Donovan J, Owen-Smith A, Potokar J, Longson D, Hawton K. et al Factors influencing the decision to use as a method of suicide: qualitative study. Br J Psych. 2010;197(4):320–5. 10.1192/bjp.bp.109.076349 . 20884956

[19] Abd-Elwahab Hassan D, Ghaleb SS, Kotb H., Agamy M, Kharoshah M. Suicidal hanging in Kuwait: retrospective analysis of cases from 2010 to 2012. J Forensic Leg Med. 2013;20 (8):1118–21. 10.1016/j.jflm.2013.09.021 . 24237833

[20] Azmak D. Asphyxial deaths: a retrospective study and review of the literature. Am J Forensic Med Pathol. 2006;27(2):134–44. 10.1097/01.paf.0000221082.72186.2e . 16738432

[21] Hunt IM, Windfuhr K, Shaw J, Appleby L, Kapur N. National confidential inquiry into suicide and homicide. Ligature points and ligature types used by psychiatric inpatients who die by hanging: a national study. Crisis. 2012;33(2):87–94. 10.1027/0227-5910/a000117 . 22343063

[22] Suárez-Peñaranda JM, Álvarez T, Miguéns X, Rodriguez-Calvo MS, De Abajo BL, Cortesão M, et al Characterization of lesions in hanging deaths. J Forensic Sci. 2008;53(3):720–3. 10.1111/j.1556-4029.2008.00700.x . 18471222

[23] Hernández-Alvarado MM, González-Castro TB, Tovilla-Zárate CA, Fresán A, Juárez-Rojop IE, López-Narváez ML, et al Increase in suicide council by hanging in the population of Tabasco, Mexico between 2003 and 2012. Int. J. Environ. Res. Public Health 2016, 13(6):552. 10.3390/ijerph13060552 PMC4924009 27258292

[24] Habenstein A, Steffen T, Bartsch C, Michaud K, Reisch T. Chances and limits of method restriction: a detailed analysis of suicide methods in Switzerland. Arch Suicide Res. 2013;17(1):75–87. 10.1080/13811118.2013.748418 . 23387405

[25] Joo SH, Wang SM, Kim TW, Seo HJ, Jeong JH, Han JH, et al Factors associated with suicide completion: a comparison between suicide attempters and completers. Asia Pac Psychiatry. 2016;8(1):80–6. 10.1111/appy.12216 . 26477349

[26] De Leo D, Dwyer J, Firman D, Neulinger K. Trends in hanging and firearm suicide rates in Australia: substitution of method? Suicide Life-Threat Behav. 2003;33(2):151–64. . 12882416 10.1521/suli.33.2.151.22775

[27] Sarchiapone M, Mandelli L, Iosue M, Andrisano C, Roy A. Controlling access to suicide means. Int J Environ Res Public Health. 2011;8(12):4550–62. . 22408588 10.3390/ijerph8124550 PMC3290984

[28] SNSF [Swiss National Science Foundation]. Suicide in Switzerland: A detailed national survey of the years 2000 to 2010. Retrieved December 12 2018, from http://p3.snf.ch/project-133070.

[29] Thoeni N, Reisch T, Hemmer A, Bartsch C. Suicide by firearm in Switzerland: who uses the army weapon? Results from the national survey between 2000 and 2010. Swiss Med Wkly. 2018;148:w14646 . 30378640 10.4414/smw.2018.14646

[30] Ruff F, Bartsch C, Glasow N, Reisch T. [Article in German; Abstract available in German from the publisher] [Suicides of psychiatric inpatients - a systematic recording in Switzerland of the years 2000 to 2010]. Psychiatr Prax. 2018;45(6):307–313. . 29665611 10.1055/s-0043-120888

[31] Gauthier S, Reisch T, Bartsch C. Swiss prison suicides between 2000 and 2010. Crisis. 2015; 36(2):110–116. . 25708254 10.1027/0227-5910/a000302

[32] Glasow N. Structural suicide prevention in inpatient psychiatric facilities. 2011 Berlin: Logos.

[33] Wu CY, Lin YY, Yeh MC, Huang LH, Chen SJ, Liao SC, Lee MB. Effectiveness of interactive discussion group in suicide risk assessment among general nurses in Taiwan: a randomized controlled trial. Nurse Educ Today. 2014, 34(11):1388–1394. 24768204 10.1016/j.nedt.2014.03.015

